# Partnering with Schools to Adapt a Team Science Intervention: Processes and Challenges

**DOI:** 10.1007/s12310-024-09665-7

**Published:** 2024-05-02

**Authors:** Aparajita Biswas Kuriyan, Jordan Albright, Samantha Rushworth, Biiftu Duresso, Shannon Testa, Ricardo B. Eiraldi, Edward W. Marshaleck, Courtney Benjamin Wolk

**Affiliations:** 1 Center for Mental Health, University of Pennsylvania Perelman School of Medicine, 3535 Market St. 3rd Fl., Philadelphia, PA 19104 USA; 2 Pediatrics and Psychiatry, University of Pennsylvania Perelman School of Medicine, Philadelphia, PA USA; 3 Children’s Hospital of Philadelphia, Philadelphia, PA USA; 4 Upper Darby School District, Upper Darby, USA; 5 Leonard Davis Institute of Health Economics, University of Pennsylvania, Philadelphia, PA USA; 6 University of South Alabama, Mobile, AL USA

**Keywords:** School mental health, Team science, Interdisciplinary collaboration, Implementation support

## Abstract

Public schools are a major provider of mental health services for children in the US. Mental and behavioral health services range from universal programming to individualized clinical supports to address student needs. These services in schools are delivered by various professionals including non-teaching and teaching school personnel, school-employed clinicians, and/or contracted community mental health partners. Provision of mental health services requires complex coordination of providers across disciplines, although few professionals have training in multidisciplinary collaboration strategies. Attention to team processes, such as delineating team members' roles, improving communication, and identifying collaboration strategies, may impact the effectiveness of evidence-based mental health service provision in real world settings. One intervention, Team Strategies and Tools to Enhance Performance and Patient Safety (TeamSTEPPS), has been used in healthcare with positive outcomes and has been adapted to educational settings. The current paper describes the community-partnered subsequent adaptation of TeamSTEPPS for schools. Needs assessment interviews identified challenges, successes, and goals for student mental health. Overarching themes extracted from interviews include limited resources at multiple levels (e.g., financial support, time, and personnel), communication challenges, and poor role clarity. A community advisory board provided guidance during the adaptation and implementation planning process. Adaptations to the intervention included tailoring the intervention to the school context and the development of flexible training plans. In addition, individualized implementation plans were developed with each school partner to mitigate foreseeable barriers to rolling-out TeamSTEPPS. Our team is currently piloting the adapted TeamSTEPPS intervention and implementation strategies in partnership with three school districts.

**Supplementary Information:**

The online version contains supplementary material available at 10.1007/s12310-024-09665-7.

## Introduction

Public schools have become the main provider of mental health services to children in the US and offer a way to improve access for all, including youth living in low-resource communities (Duong et al., 2021). In fact, rates of mental health service utilization in schools rival outpatient settings and surpass other settings such as primary care and juvenile justice (Costello et al., 2014; Duong et al., 2021; Kamali et al., 2022). A 2017–2018 national survey shows that 55% of schools provided mental health diagnostic services both in and outside the school setting (Wang et al., 2020). Most school mental health services in the US are delivered through contractual partnerships with community mental health organizations (Reaves et al., 2022). Students with mental health and behavioral concerns commonly receive services in schools along with additional services in other settings, highlighting the need for coordination among different sectors (Farmer et al., 2003).

### Challenges with Mental Health Service Provision in Schools

Although providing services within the school setting improves the reach of behavioral health interventions, it can be a complex array of services that are challenging for administrators and school staff to understand and utilize effectively. The structure of mental health services in school varies greatly and requires coordination among administration, teaching staff, counseling staff, and community providers (Whitaker et al., 2018). The concept of multi-tiered systems provides some conceptual organization to a bevy of services which, at its best, is a coordinated system of integrated services with specified inclusion criteria, referral patterns, and outcomes achieved, and at its worst is a disorganized and misused system of services (Fabiano & Evans, 2019). Overall challenges in school mental health provision are attributed to numerous barriers including limited time for collaboration among teachers and mental health providers, competing demands, poor communication, and vaguely defined roles (Bates et al., 2019; Holmesland et al., 2010; Mendenhall et al., 2013; Odegard, 2005; Zabek et al., 2023).

In addition to structure, the quality of mental health services in schools varies greatly as evidence-based programs have low adoption rates overall (Hoover, 2018; Kilbourne et al., 2018). Some challenges related to evidence-based program implementation in schools include a mismatch in resources required to implement programs versus available resources, low motivation from practitioners to implement evidence-based programs, attitudes of school personnel related to prioritizing scientific research in educational settings, and lack of organizational processes in districts to support implementation of EBPs (Hoover, 2018; Olson et al., 2021; Whitaker et al., 2018).

### Cross-sector Collaboration in School Mental Health

Funding streams, the availability of community resources, and other factors impact who will provide mental health services in schools, which leads to a potentially wide variety of staff who may be responsible for children’s mental health in schools. Furthermore, school mental health professionals (e.g., social workers, school psychologists, and counselors) are often responsible for duties not related to social, emotional, or behavioral health, such as scheduling courses and administrative paperwork (Zabek et al., 2023). This results in a complex dynamic of roles devoted to mental/behavioral health-related programming, and the risk for role confusion and underutilization of mental health expertise is high. For example, teachers are often the first to notice a concern but may not be aware of resources available to the student or when to involve other staff members (Dimitropoulos et al., 2022). To complicate matters further, school personnel experience challenges collaborating with community professionals who provide services in schools. For example, understanding legal guidelines around confidentiality, role confusion, and limited time to collaborate are cited as common challenges (Weist et al., 2012). Schools may have a clear structure for mental health service provision, but not all staff members are aware of how to effectively refer students to services. Without a clearly communicated plan for mental health service provision, a host of problems is bound to arise including underserved students, duplication of services, or untimely service provision.

On the other hand, numerous advantages of cross-sector collaboration have been demonstrated for students and school staff. Schools who collaborate with community mental health organizations provide more mental health supports, such as school-wide social-emotional learning programs and wellness committees, than schools who do not collaborate with CMHCs (Williams et al., 2018). School staff also report reduced burnout, increased knowledge of community resources, and increased confidence in handling mental health concerns (Heatly et al., 2023; Williams et al., 2018). Studies demonstrate that improved collaboration improves student attendance, reduces disciplinary referrals, improves academic achievement, and improves behavioral health outcomes (Bates et al., 2019; Splett et al., 2017). An intervention that provides specific strategies for improving collaboration and communication across multiple partners offers a potential solution to improving cross-sector collaboration in school mental health, which will in turn improve school mental health service provision.

### Team Science Informed Interventions

Team Strategies and Tools to Enhance Performance and Patient Safety (TeamSTEPPS; Agency for Healthcare Research and Quality, n.d.) is an evidence-based intervention that has been widely disseminated in healthcare sites globally (Mahoney et al., 2012; Mayer et al., 2011) and more recently adapted for additional settings (e.g., child advocacy; McGuier et al., 2023), including schools (Wolk, et al., 2019a). Initially designed for healthcare settings, TeamSTEPPS is grounded in evidence from other fields such as human factors theory, aviation, change management, and the military, which helped identify key competencies to support successful teamwork (King et al., 2008). TeamSTEPPS is designed to build competencies in the areas of leadership, situation monitoring, mutual support, and communication and has been associated with improvements in teamwork and communication as well as patient outcomes (King et al., 2008; Mahoney et al., 2012; Sheppard et al., 2013; Stead et al., 2009). Building on TeamSTEPPS’s long history of success in a variety of healthcare settings, adaptation to additional settings is nascent. A recent pilot adaptation of TeamSTEPPS for child advocacy centers demonstrates the potential of team training to increase knowledge of shared goals and improve shared awareness, communication, and perspective-taking (McGuier et al., 2023).

The Consolidated Framework for Implementation Research (CFIR; Damschroder et al., 2009) provides an overarching framework to guide implementation. The CFIR, synthesized from the health services implementation literature, represents “an overarching typology—a list of constructs to promote theory development and verification about what works where and why across multiple contexts” (Damschroder et al., 2009). Guided by CFIR, the research team chose TeamSTEPPS due to intervention characteristics that are favorable for implementation in school settings. Out of the five major domains of the CFIR, the current study focuses on the inner setting, which consists of structural characteristics, networks and communication, culture, implementation climate, and readiness for implementation. The TeamSTEPPS training is designed to be adaptable to different settings (e.g., office-based and hospital), audiences (e.g., students, physicians, leadership, and administrative staff), and training constraints in that the curriculum does not dictate a specific length or sequence for the modules. TeamSTEPPS provides conceptual guidelines and practical strategies through four core modules aligned with the core competencies based in the team science literature and allows for adaptation to specific content (Chen et al., 2019). The TeamSTEPPS curriculum, including all supporting materials, is freely available online (https://www.ahrq.gov/teamstepps-program/index.html).

Other commonly implemented programs in schools, such as Multi-Tiered Systems of Support (e.g., Positive Behavior Interventions and Supports; PBIS) or learning collaboratives, also attend to team processes but in different ways. The main way in which TeamSTEPPS differs is that it was designed to be a modular and potentially efficient method for improving teamwork practices. TeamSTEPPS is complementary to these other approaches and offers service providers a conceptual framework that can be flexibly applied, as well as practical tools to improve teamwork practices and enhance the efficiency of service delivery. TeamSTEPPS provides tools that can transform leadership and support collaboration, communication, and conflict-resolution, providing a foundation for school mental health teams to strengthen their service provision, including, but not limited to, MTSS implementation.

### Initial TeamSTEPPS School Mental Health Adaptation

The purpose of the first pilot adaptation of TeamSTEPPS (Wolk, et al., 2019a) was to establish preliminary feasibility and acceptability of TeamSTEPPS for school mental health clinicians. The adaptation was informed by the school mental health teams employed by community organizations; school-employed staff did not participate (Wolk, et al., 2019a). Community-employed school mental health teams participated in an advisory board that met monthly for 5 months to recommend adaptations to the TeamSTEPPS intervention (see [Wolk, et al., 2019b] for a full description of the advisory board and activities). The adaption resulted in revisions in language that is consistent with the preferred nomenclature in schools (e.g., “patients” was changed to “students” or “children”) and revised case examples informed by school/community partner experiences. Core TeamSTEPPS content remained largely unchanged in the adaptation because participants reported it was relevant as is.

School mental health teams were subsequently randomized to participate in a 4-h in-person workshop of the adapted TeamSTEPPS or continue delivering services as usual. Qualitative results supported feasibility and acceptability of TeamSTEPPS according to community-employed mental health professionals, but no difference in teamwork attitudes and perceptions were observed post-intervention compared to the control group (Wolk, et al., 2019a). Interviewees indicated acceptability of TeamSTEPPS by expressing positive perceptions of the training and an appreciation for a focus on communication (Wolk, et al., 2019a). Feasibility was also noted by interviewees due to the ease of use, reasonable time-commitment, and high potential for integrating into daily workflows (Wolk, et al., 2019a). This is consistent with findings from another study that adapted TeamSTEPPS for child advocacy. The adapted TeamSTEPPS training was found to be acceptable, feasible, and appropriate on average, with ratings close to or surpassing 4 on a 5-point scale (McGuier et al., 2023). Follow-up interviews from the school-based pilot study suggested that additional modifications would be helpful to maximize appropriateness for the school context and address inter-organizational challenges between mental health and school personnel (Wolk, et al., 2019b). Specifically, engaging school personnel directly in further adapting TeamSTEPPS to better support teaming between mental health professionals and school staff was highlighted as a needed next step. Feedback also indicated that leadership involvement was an important facilitator to implementation but additional robust supports, such as ongoing consultation and booster training, would enhance implementation efforts. This is in line with other research that shows that consultation after training enhances implementation outcomes (Lyon et al., 2022).

## Current Study

The current manuscript reports on the first two aims of a 5-year ongoing hybrid quasi-experimental study with descriptive information about the process of adapting TeamSTEPPS in collaboration with school mental health teams and other school personnel. At the time of writing the current article, the larger trial is in its 4th year. Additional details about the larger study, including the conceptual framework guided by CFIR, can be found in the published study protocol (Kuriyan, et al., 2021).

The first aim of the study is to capture pre-implementation information about current and historical use of school mental health services from our study school partners in order to provide context for efficient collaborative adaptation of the TeamSTEPPS intervention. We utilize interview and observational data to summarize participant perspectives of challenges and successes implementing school mental health services, along with teamwork-related variables that hinder or support school mental health. The second aim is to adapt TeamSTEPPS and develop a corresponding tailored implementation plan. Complementary to CFIR, the School Implementation Strategies, Translating ERIC Resources (SISTER; Cook et al., 2019) is a guide for tailoring implementation strategies adapted from the Expert Recommendations for Implementing Change (ERIC) taxonomy, specifically for schools (Cook et al., 2019). We describe our process for adapting TeamSTEPPS and developing tailored implementation plans, using SISTER as a guide, for each school entity we partnered with.

## Method

All study procedures were approved by the University of Pennsylvania’s Institutional Review Board. Informed consent was obtained from participants prior to engagement in research activities.

### Participants

Our research team partnered closely with three distinct school entities in or surrounding one major metropolitan area in the Mid-Atlantic region of the US. School partners ranged from a single charter school to a district comprised of 15 schools, as described in Table 1.

**Table 1:** Student demographics of partner districts for the 2021–2022 school year

Characteristics	Partner 1	Partner 2	Partner 3
	*n* (%)	*n* (%)	*n* (%)
Students enrolled	12,395	2283	443
Schools in district	15	3	1
Total teachers	801	170	31
Average years of experience	12.5	–	6.3
Race/ethnicity			
Black/African American	5952 (48.0)	301 (13.2)	338 (76.3)
White	2373 (19.1)	1396 (61.1)	–
Asian	1821 (14.7)	122 (5.3)	0 (0.0)
Multi-racial	1649 (13.3)	61 (2.7)	–
Hispanic	579 (4.7)	390 (17.1)	44 (9.9)
American Indian	0 (0.0)	8 (0.4)	–
Native Hawaiian/Pacific Islander	0 (0.0)	6(0.3)	0 (0.0)
Unknown	21 (0.1)	0 (0.0)	61 (13.8)
Gender			
Female	6010 (48.4)	–	218 (49.2)
Male	6385 (51.5)	–	225 (50.8)
Eligible for reduce/free lunch (%)	30.06–84.92	49	100

Omitted data were not reported by partner districts. Data for teachers are from (1) “2021–2022 Professional Staff Summary” by the Pennsylvania Department of Education, n.d.a. (https://www.education.pa.gov/DataAndReporting/ProfSupPers/Pages/ProfStaffSummary.aspx). Copyright 2024 by Commonwealth of Pennsylvania and (2) “2021–2022 Certified Staff” by the New Jersey Department of Education, n.d.a. (https://www.nj.gov/education/doedata/cs/). Copyright 1996–2020 by the State of New Jersey. Student demographics data were retrieved from (1) “2021–2022 PA Lunch Report” by the Pennsylvania Department of Education, n.d.b. (https://www.education.pa.gov/Teachers%20-%20Administrators/Food-Nutrition/reports/Pages/National-School-Lunch-Program-Reports.aspx). Copyright 2024 by Commonwealth of Pennsylvania; (2) “Public School Enrollment 2021–2022” by Pennsylvania Department of Education, n.d.c. (https://www.education.pa.gov:443/DataAndReporting/Enrollment/Pages/PublicSchEnrReports.aspx). Copyright 2024 by Commonwealth of Pennsylvania; and (3) “2021–2022 Enrollment District Reported Data” by the New Jersey Department of Education, n.d.b *(*https://www.nj.gov/education/doedata/enr/). Copyright 1996–2020 by the State of New Jersey

Schools were invited to participate in the research study via an introductory email sent to district and/or school leadership. The research team utilized public data sources to identify school districts in the metropolitan area that served primarily low-income and/or historically minoritized populations and emailed and/or called the district representative for student services. Interested school partners provided a letter of support documenting interest and understanding of the expectations of participation. Following approval from school leadership, individual staff members were invited to participate in needs assessment interviews via an email sent by study staff. Select participants in leadership roles in relevant community organizations (e.g., payors) were also invited to participate in interviews and recruited directly by study staff via email.

Twenty-two participants were interviewed for the needs assessment (Table 2). Participants identified as predominantly female (*n* = 20; 90.9%), White (*n* = 20; 90.9%), and non-Hispanic/Latinx (*n* = 21; 95.5%). Participants included school leaders (*n* = 5; 22.7%), mental health staff (*n* = 7; 31.8%), teachers and counselors (*n* = 6; 27.3%), and community mental health personnel (*n* = 4; 18.2%).

**Table 2:** Demographic characteristics of interview and advisory board participants

Characteristic	Interview	Advisory board
	*n* (%)	*n* (%)
Total participants	22	15
Gender		
Female	20 (90.9)	–
Male	2 (9)	–
Race		
White	20 (91)	–
Black	2 (9)	–
Ethnicity		
Hispanic/Latinx	1 (4.5)	–
Non-Hispanic/Latinx	21 (95.5)	–
Participant roles		
School leadership	5 (22.7)	5 (33.3)
School mental health staff	7 (31.8)	5 (33.3)
Teachers and other school staff	6 (27.3)	5 (33.3)
Community collaborators	4 (18.2)	0
Participant affiliation		
Partner 1	4 (18.2)	5 (33.3)
Partner 2	11 (50.0)	5 (33.3)
Partner 3	4 (18.2)	5 (33.3)
Community organizations	3 (13.6)	0 (0.0)

Key staff from our partner districts identified individuals (*n* = 15) to participate in our advisory board (see Table 2). We requested that the advisory board be comprised of individuals from different groups, such as one to two members each from the leadership team, teaching staff, student service team members, and mental health staff. The 15 individuals invited to join the advisory board spanned a range of leadership (e.g., assistant superintendents), clinical (e.g., school social workers, psychologists, and counselors), and educational (e.g., grade teacher and special education liaison) roles in schools. Each school partner had five individuals in attendance.

### Measures

A semi-structured guide was developed to support the needs assessment interviews, informed by our prior TeamSTEPPS work, that included questions and prompts to query about (1) the history of school mental health services in the interviewee's district, (2) successes, and (3) challenges or unintended consequences of previous efforts. Additionally, we inquired specifically about team functioning concepts relevant to TeamSTEPPS, such as communication (e.g., how do staff members share information), collaboration (e.g., how do mental health clinicians give recommendations), role clarity (e.g., how well do staff understand their roles), and leadership (e.g., how does leadership support implementation of recommended strategies from mental health clinicians). We also asked about current processes and strategies that may support or hinder teamwork.

### Procedures

The first aim of the study was to capture a comprehensive picture of the successes and challenges with implementing school mental health services from diverse participants. Data collection methods included needs assessment interviews and observations, which occurred between December 2020 to October 2021. We conducted individual hour-long needs assessment interviews with both school-employed and community-employed participants (e.g., teachers, school mental health professionals, and administrators) regarding challenges related to school mental health service integration and collaboration. We also attended school mental health team meetings in our partner schools, typically virtually as necessitated by the COVID-19 pandemic. To inform future TeamSTEPPS adaptations and implementation planning, research team members took field notes during the meeting about the observed agenda, contributions of staff members (e.g., how many people spoke during the meeting, who offered solutions, who appeared to lead), tasks accomplished, time spent discussing student mental health (e.g., intervention planning and problem solving for difficult cases) relative to administrative tasks (e.g., documenting student attendance, paperwork), and levels of support provided to staff (e.g., time for praise/recognition).

For the second aim of the study, we utilized a community partnership model to collaborate with an advisory board to develop and adapt TeamSTEPPS team training and implementation strategies for school mental health teams to improve care (Southam Gerow et al., 2009). Before participating in the advisory board meetings, potential participants completed a form that outlined the mission, guidelines, and expectations of the meeting. Participants indicated their goals for participation, special interests or experiences they could offer, and questions they had ahead of the meeting, and then signed and dated the form. We offered flexible scheduling options for the advisory board via a survey and communication with school leadership, and ultimately, schools decided that two 4-hour meetings were most feasible. Meetings were held virtually in January 2022 due to the COVID-19 pandemic. Demographic characteristics of advisory board participants were not collected as they were considered members of the research team and not study participants.

The agenda for the advisory board meetings was developed with the knowledge we had obtained from the needs assessment interviews and observation in mind and is provided in the supplemental materials. At the first meeting, we briefly reviewed the core components of TeamSTEPPS and solicited recommended adaptations for the school context. Next, we asked about potential barriers and facilitators to implementing TeamSTEPPS with school mental health teams, and identified possible adaptations so that its applicability to teams would be enhanced. The goal of the advisory board meetings was to work toward an adapted TeamSTEPPS that incorporates both school mental health and school-employed personnel as team members, and specific, tailored implementation strategies to support TeamSTEPPS implementation.

At the second and final meeting, we focused on collaborative implementation planning to ensure that TeamSTEPPS could be successfully implemented and sustained after the study. Advisory board members participated in small-group activities with their own school team facilitated by one to two members of the research team. The first small-group activity consisted of listing and categorize potential barriers and the perceived impact of those barriers to implementing TeamSTEPPS. This activity serves to illustrate the process undertaken to move from soliciting information to identifying and prioritizing implementation supports in partnership with school collaborators. Categorizing barriers helped the group focus on which barriers to prioritize when thinking about implementation support strategies that might mitigate those barriers. The second small-group activity entailed brainstorming potential solutions to key barriers. The meeting culminated in a large-group activity that involved sharing and discussing the results of the small-group work. This included discussing the implementation process, identifying primary champions of TeamSTEPPS, and incorporating tailored examples of strategies within the training materials.

Information from the needs assessment interviews, observations, and community advisory board activities were utilized by the research team to complete adaptations to TeamSTEPPS and develop tailored implementation plans. Following the final advisory board meeting, our team determined additional perspectives were needed before finalizing adaptations. We then met with the advisory board members from each school partner group (one 60-min meeting per school entity) to check that we had adequately addressed their input with our adaptations and to collaboratively finalize implementation plans.

#### Analytic Plan

We utilized a rapid analysis procedure for the needs assessment interviews and field notes, which has the advantage of providing timely and actionable results when planning for implementation and has been shown to produce descriptive results that are consistent with traditional in-depth qualitative coding (Taylor et al., 2018). Interviewers took detailed notes during interviews. Then, a member of the research team synthesized the key themes from interviews and field notes across each district entity following a line-by-line review of interview and field notes. Themes and district summaries were reviewed by the study interviews and observers to ensure accuracy. We engaged in member checking during interviews using clarifying probes and summarization, as well as with our district partners, such as during advisory board meetings where we summarized the themes of the needs assessments and asked for reflections, additional input, and validation.

## Results

### Pre-implementation Needs Assessment

Across needs assessment interviews, several themes were found to impact team functioning. These fell into three broad categories, including (1) intra- and inter-organizational challenges, (2) inter-organizational successes, and (3) future team goals.

#### Intra- and Inter-organizational Challenges

Across all interviews, limited resources (i.e., time, financial support, and personnel) were identified as a challenge to the successful provision of mental health services in schools. Interviewees described having insufficient time to complete job-related tasks, which was attributed to several causes. School mental health providers who were contracted from community mental health agencies to provide services in schools reported typically being onsite only when engaging in billable hours, which they described as limiting their ability to have an impact in their schools. Additionally, participants reported that funding shortages have resulted in the dismissal of support staff, thereby creating greater demands for existing team members. Low staffing levels are reportedly further exacerbated by other challenges, such as having a large student body, which creates an increased demand on an already stressed, underfunded system.

Several interviewees shared examples of how poor role clarity can lead team members to take on responsibilities outside of their scope, leading to poor intra-organizational collaboration. One school staff member shared that many members of their team are asked to record student attendance, manage class scheduling, and perform hall monitor duties. Another individual shared this concern, indicating that there is an attitude of, “anyone can do anything,” within their district. Interviewees shared that school administrators are asked to prioritize issues such as attendance or academic performance, thereby encouraging school staff to fulfill roles that attend to these concerns (e.g., track attendance for truancy cases) which takes away time from student mental health-related responsibilities. It was noted that, at face value, this seems like a highly collaborative and supportive mentality; however, interviewees reported that this approach only further decreases the clarity of roles. Participants emphasized that this approach only attends to symptoms of a larger issue (e.g., student mental health, poverty, and trauma) which may be effectively addressed through the provision of adequate mental health services. When asked why staff and administrative personnel choose to hand off these responsibilities to school mental health providers, interviewees hypothesized that this may be due to a lack of prioritization of mental healthcare or a lack of understanding regarding team members’ roles.

A final shared concern among all interviews was related to the inefficient use of meeting time. Staff emphasized that internal team meetings and external meetings with contracted mental health service providers are often overwhelmed by administrative tasks (e.g., discussing attendance and completing spreadsheets) to the detriment of case discussion. As a result, meetings run over time or discussions are rushed to end on time. A need for more clearly delineated meeting agendas with explicit goal setting at the beginning and distribution of action items at the end was expressed. Participants shared examples of how poor communication between team members, administration, and teachers resulted in school mental health providers being left out of important discussions regarding students’ mental health needs and decisions regarding disciplinary actions for students on their caseloads. One group also shared that communication challenges were common when attempting to collaborate with the contracted school-based mental health agency onsite. The need for more systematic improvements in communication inside and outside of meetings was an expressed need of many participants.

Several additional challenges mentioned include a fear of being written up by their superiors, which stifles assertiveness and advocacy in team meetings. Other teams noted that teacher stress can be a challenge to collaboration. Others explained that certain rules around confidentiality can make discussing cases and collaborating with teachers or other providers difficult.

#### Inter-organizational Successes

All interviewees shared that they had good success engaging in inter-organizational collaboration with outside behavioral health and mental healthcare agencies. Participants reported that through partnerships with outside agencies, students are better able to access services, including services for behavior challenges or crisis situations. Several community partners emphasized that these partnerships are particularly successful when the outside agency has a physical presence in the school building or on school grounds.

#### Team Goals

All interviewees expressed the goal of increasing access to and use of mental health services for children and families in their community. Many indicated that this goal was particularly important for the students and families in the district who are from low socioeconomic, racially minoritized backgrounds, who may experience challenges accessing mental healthcare otherwise due to barriers such as cost or stigmatization. Additionally, substance use, community violence and trauma were commonly identified mental health-related concerns within the communities of our partner schools. As such, interviewees emphasized the importance of accessible mental health services that families can navigate to address these challenges.

Participants alluded to the connection between student mental health and academic performance. Specifically, they indicated that by improving access to mental health services in the school, they hope to improve student mental health but also academic performance as a byproduct.

### Intervention Adaptations

The results of the advisory board’s collaborative effort to identify adaptations needed to the TeamSTEPPS training protocol are organized according to the Framework for Reporting Adaptations and Modifications-Expanded (FRAME; Stirman et al., 2019). Key modifications to the training content included identifying and specifying concrete strategies for improving communication between mental health professionals and school personnel, clarifying roles (e.g., around classroom behavior management and crisis care), strategically engaging leaders from both schools and mental health agencies in team processes, and leveraging existing educational meetings more efficiently to collaboratively support student mental health. We also identified that the Situation Monitoring module of TeamSTEPPS could be subsumed within Mutual Support and Communication strategies given the nature of situational monitoring activities that could be accomplished in a school setting. We also identified necessary revisions to the TeamSTEPPS training materials, including developing case scenarios specifically tailored to the context of school mental health services and adding a “working meeting” portion to the training where training participants work with study staff to begin customizing TeamSTEPPS strategies and materials to fit the needs of their respective teams.

All adaptations were made either in direct partnership with participants or guided by feedback from them. Additionally, all adaptations preserved the core functions and critical components of the original TeamSTEPPS training.

### Implementation Planning

Table 3 lists key barriers that were identified during the advisory board illustrated by examples obtained during the advisory board and interviews, and how the barrier was addressed in the adapted intervention/tailored implementation plan. Table 4 lists and describes preparation and ongoing support strategies selected by advisory board members for successful implementation of TeamSTEPPS, which is organized using terminology from the SISTER guide (Cook et al., 2019). Although certain strategies were common across all schools, the actual plan for those strategies varied from school to school. For example, our team offered schools the ability to structure TeamSTEPPS training according to their staffing needs and professional development schedule. Educational materials developed for each school were similar in that there was a set of standard materials such as slides and handouts, but some schools requested customized materials targeted to their areas of development and need, such as a handout on communicating with families using TeamSTEPPS principles and a customized agenda outline for child study team meetings.

**Table 3:** Key barriers and solutions

Barrier	Advisory board and interview examples of barrier	Approach to addressing barrier in TeamSTEPPS training and/or implementation plan
Lack of communication and coordination between mental health and education personnel	Interviewees noted siloing and lack of shared record systems limit information sharing between mental health staff and teachers	Incorporated examples of communication breakdowns between mental health and education staff in trainings and explicitly discuss how TeamSTEPPS strategies can address them
Leadership disengagement	Interviewees and advisory board participants expressed concern that, in some schools, principles and relevant administrators may not engage in TeamSTEPPS training	Emphasized during TeamSTEPPS training the concept of shared leadership; consultation in some schools/districts to support teams in engaging leaders; developed a brief, targeted TeamSTEPPS training for principals to reduce burden of participating
Team composition is fluid	During advisory board meetings, some participants expressed concern about how to implement TeamSTEPPS in teams where the team membership shifts day to day (e.g., because some districts have mental health staff working in multiple schools) or where roles sometimes overlap	Clarify/emphasize during training how TeamSTEPPS can be applied in teams with fluid membership; added information about role clarity to the Team Structure section and relevant student service team examples (e.g., classroom behavior management, crisis care); added discussion to the training about how leaders can create systems to account for shifting membership (e.g., by standardizing checklists and procedures)
Competing priorities/lack of time	During interviews, participants discussed innovation fatigue and how new programs have historically faded out once the initial training/active support phase ends. Advisory board participants discussed that professional development time is limited and that it could be difficult to fit TeamSTEPPS training in	Engaged leaders early in planning for sustainment. Developed a flexible, modular training plan for TeamSTEPPS to accommodate different district professional development schedules
Burnout	During advisory board meetings, some participants expressed that staff are burnt out in the aftermath of the COVID-19 pandemic and that this might dampen enthusiasm for new programs like TeamSTEPPS	Incorporated how TeamSTEPPS mutual support strategies can mitigate staff burnout into the training
Staff turnover	During advisory board meetings, participants emphasized high staff turnover and the importance of planning proactively for that	Developed TeamSTEPPS training materials with sustainability in mind, such as by ensuring training slides had complete speaker notes and that handouts were very detailed so that they could be transferred to district personnel; worked with each district to identify internal implementation leads; developed a detailed TeamSTEPPS implementation guide for district leaders; devised plan to create asynchronous training content for districts to share with new staff during onboarding
Medical terminology is off-putting to school staff	During advisory board meetings, participants shared the importance of the TeamSTEPPS training feeling relevant and specific to the school context	Revised TeamSTEPPS language to reflect preferred nomenclature in schools (e.g., replaced “patients” with “students” or “children”) rather than healthcare, including case examples

**Table 4:** Collaboratively developed tailored implementation plan components by school partner

SISTER implementation strategies	School partner 1	School partner 2	School partner 3
Tailor [intervention] strategies^a^	TeamSTEPPS materials tailored to context (e.g., to reflect staff titles/roles and organizational structure)	TeamSTEPPS materials tailored to context (e.g., to reflect staff titles/roles and organizational structure)	TeamSTEPPS materials tailored to context (e.g., to reflect staff titles/roles and organizational structure)
Conduct ongoing training	First, two small trainings with individual school service teams Next, one training with all student service staff Training for higher level leaders	One two-part training for all student service staff	One training for all student service staff
Provide ongoing consultation	Monthly meetings with leadership	Scheduled as needed	Scheduled as needed
Develop educational materials	TeamSTEPPS strategy handouts The google drive was comprised of all the materials that were developed for the other two schools	TeamSTEPPS strategy handouts Agenda template for student service team meetings Family handout on common IEP questions Staff guide for using SBAR^b^ for both verbal and email communication	TeamSTEPPS strategy handouts Summary of information about electronic health record systems for school mental health staff Guide for families on what questions to ask when receiving services Staff guide on confidentiality rules around student mental health
Distribute educational materials	Email, Google Drive	Email to key personnel to distribute to other staff	Email to key personnel to distribute to other staff
Use advisory boards and workgroups	During pre-implementation to inform adaptation and implementation planning Ongoing monthly meetings between research team, assistant superintendent, and director of pupil services to plan for sustainment and spread across district	During pre-implementation to inform adaptation and implementation planning	During pre-implementation to inform adaptation and implementation planning
Stage implementation scale up	Staged training groups, then ongoing collaboration with district leaders to support scale up and sustainability	No specific scale up plan—all eligible staff trained concurrently	No specific scale up plan—all eligible staff trained concurrently

^a^All intervention strategies were tailored to meet the needs and preferences of each school partner while retaining fidelity to the intervention. Notable tailoring is denoted in Table 2 for each strategy when applicable

^b^SBAR (Situation, Background, Assessment, Recommendation) refers to a TeamSTEPPS strategy for communicating information to team members concisely

## Discussion

Although school administrators and staff agreed that social, emotional, and behavioral problems are a high priority for schools to address (Briesch et al., 2020), the reality of providing comprehensive and timely mental health services for students has challenged the field. The current study reported descriptive results from the first 2 years of an ongoing hybrid quasi-experimental study of TeamSTEPPS, a team science intervention for improving collaboration and communication skills, adapted for school mental health teams. In using the CFIR as a framework to guide study design, we were able to prospectively identify implementation challenges, adapt the intervention, and tailor implementation procedures prior to large scale roll-out as recommended (Kirk et al., 2016). Results from the current study described procedures for adapting the TeamSTEPPS intervention in collaboration with school partners and are discussed below as lessons learned.

### Lessons Learned 1: Relevance of Team Science Constructs to School Mental Health

Needs assessment interviews with multiple community and school partners were critical for gauging current challenges, goals, and successes for schools regarding mental health programming. Our prior work with community-employed school-based mental health professionals spoke to the potential relevance of teamwork variables in the provision of school-based mental health. Our current work with a larger and more varied subset of school professionals echoed the same sentiment. Challenges related to limited resources, unclear roles, and communication barriers have also been highlighted in other research studies (Bates et al., 2019; Holmesland et al., 2010; Mendenhall et al., 2013; Odegard, 2005). Operating with limited resources is a stark reality for most public schools in the US, which has an impact on other barriers such as role confusion. For example, in a recent review exploring the time allocation of mental health staff in schools, researchers found that these staff are often underutilized for supporting student mental health due to competing demands on their time for non-mental health-related tasks (Zabek et al., 2023). In light of ongoing workforce shortages in children’s mental health, various strategies have been proposed to mitigate the impact of these shortages, including the increasing responsibility of the educational sector in providing mental health services across the continuum. School mental health services require a team approach in that services are often provided by non-mental health trained professionals (task-shifting; McQuillin et al., 2019), but perhaps attention also needs to be given to how time is allocated to mental health trained staff.

Cross-sector collaboration in youth mental health has resulted in numerous benefits to students, their families, and school staff. Tuning into practices that support multidisciplinary collaboration is a viable strategy to bolster a system with high need and limited resources. Our participants affirmed the benefits of working with community mental health providers, but also cited barriers to finding time for effective collaboration. Other studies have shown that interventions to improve cross-sector collaboration resulted in district-wide improvements in applying evidence-based mental health practices (Connors et al., 2020; Heatly et al., 2023), underscoring the importance of further research in this area. Given the “all hands on deck” approach to providing mental health services in schools, it was no surprise that teamwork variables have been identified as important malleable constructs that impact effective service delivery.

### Lessons Learned 2: Pre-implementation Activities Prioritize Malleable Targets for Change

The needs assessment interviews, observations, and advisory board allowed our research team to better understand the context of each school partner. The needs assessment interviews provided valuable information about the implementation context for the research team. This information was useful in preparing the advisory board meeting agenda. For example, the needs assessments illuminated important differences in school mental health team organization, which influenced decisions about which activities could be accomplished during advisory board meetings as a larger group and which would be best facilitated during smaller breakout groups with each individual school entity to account for district differences. The needs assessments also helped the research team to effectively facilitate advisory board meetings. For example, insights into the culture and interpersonal dynamics of the teams informed decisions about which research team member would be the best fit for facilitating each small-group meeting. We were able to identify opportunities to make specific small changes in frequently occurring processes, which if changed, may have a compounded positive impact over time. Our partner interviews indicated that although most schools had regular meetings to discuss struggling students, there was a desire to build in time for staff to collaborate on and discuss intervention plans. Similarly, Bohnenkamp et al. (2023) found that during a learning collaborative designed to improve teaming, there was a desire to improve the effectiveness of meetings. Simply holding meetings may not be enough for effective collaboration; careful attention to each member's role and objective, and skilled facilitation of the meeting may be necessary (Hjörne & Säljö, 2014; Reuterswärd & Hylander, 2017).

### Lessons Learned 3: Include Leadership in Collaborative Adaptation

The advisory board was a way for our research team to engage in collaborative adaptation, which has shown to improve the odds of and increase readiness for implementation of evidence-based practices (Brookman-Frazee et al., 2020; Whitaker et al., 2018). Provider attitudes about the intervention, motivation to learn new skills, and challenges related to implementation are factors that impact the success of implementation (Brookman-Frazee et al., 2020). Our advisory board included all levels of school and district staff and provided structured time to discuss challenges related to different aspects of the intervention. In fact, one of the school partners tailored their implementation plan by allocating district leadership time to monthly meetings with the research team and arranged for a focused workshop on TeamSTEPPS leadership strategies. Our research team provided support in problem solving the challenges and using the flexible format of the intervention to adapt it to their needs.

Echoing our prior pilot study (Wolk, et al., 2019b), leadership emerged as an important facilitator for implementation. Our inclusion of district and school-level leadership was likely critical to engaging school staff for the duration of the study, especially during the COVID-19 pandemic. In the current sample, district leadership and school-level leadership supported implementation of TeamSTEPPS by providing staff time for training during existing professional development days, participated in team meetings, and facilitated recruitment for the advisory board. Other studies have shown that manipulating individual level factors may not be sufficient for evidence-based program adoption in schools and that leadership also needs to be engaged, which is consistent with research on the inner setting domain of CFIR (Aarons et al., 2017; Brookman-Frazee et al., 2020; Locke et al., 2020; Lyon & Bruns, 2019). The strategies in TeamSTEPPS were meant to strengthen interrelationships within teams including strategies to address leadership dynamics at multiple levels.

### Lessons Learned 4: Flexible Implementation Tools and Processes are Key to Efficient Adaptation

The adaptability of an intervention has been shown to be important for school-based mental health program implementation (Richter et al., 2022). TeamSTEPPS as an intervention possessed features such as modularity and adaptability could be implemented at low-cost and had conceptual relevance to numerous service contexts. Adaptations that were made to TeamSTEPPS in the current study included tailoring contextual content while preserving core strategies and updating the language to reflect the school setting. Adaptations to the training structure included providing flexible options to deliver training content such that each school partner could customize to their own staffing and scheduling needs to work within their available professional development time and within the parameters of the school calendar. We learned that our partner schools differed with their desired timing, pace, and level of support needed from the research team for implementing TeamSTEPPS.

The importance of improving student mental health through school-based supports and community mental health collaboration was clear in the current study. Even amidst the challenge of the COVID-19 pandemic, our partner schools remained engaged and focused on setting goals to improve student mental health. The research team’s flexibility in working with partners to implement in stages and adapt to their needs may have contributed to the persistence of the partnership despite challenges of conducting school-based research during the COVID-19 pandemic. Other research has corroborated that implementation team flexibility, rather than a prescribed "one-size fits all" implementation plan, may be an important factor in improving the uptake of an intervention in a school setting given that each school may differ on the support desired (Smith et al., 2022). The research team’s approach to adapting TeamSTEPPS for a school setting was consistent with common recommendations for adapting existing evidence-based interventions for new settings with collaborator involvement at its core (e.g., Kilbourne et al., 2007; Moore et al., 2021). Future studies should compare the effectiveness of standard implementation procedures versus tailored implementation procedures. For example, while greater pre-implementation planning is more costly, those costs may be justified if they promote implementation success.

The multi-year approach to adapting TeamSTEPPS in stages was consistent with research demonstrating that longer pre-implementation duration allows schools adequate time to thoughtfully tailor the intervention to their own organizational context and that pre-implementation activities are correlated with program implementation (Frank et al., 2022). Although TeamSTEPPS was originally designed to directly impact team functioning in high-risk occupational settings, it also has the potential to be utilized as an adjunctive intervention to support implementation of evidence-based programs. The TeamSTEPPS intervention is well-suited to guide pre-implementation activities by providing guidance on planning for team processes that impact implementation of evidence-based mental health programming such as clarifying roles and duties of those involved in implementation, establishing communication standards and expected communication patterns, and outlining a plan for high-risk situations.

### Limitations

The strengths of the study must be considered alongside its limitations. Although feedback from diverse school staff and community mental health partners was solicited, we had limited input from community mental health organizations and no input from youth or families that schools serve. With every iteration of the TeamSTEPPS adaptation, the research team has sought to increase engagement with additional collaborators. We hope that in future iterations, additional partner input can be incorporated. The current study also had limited generalizability as all schools recruited were located in under-resourced metropolitan communities.

### Future Directions

All three school partners continue to be actively engaged in the study. In the final phase of this study, our goal is to explore the feasibility, acceptability, and impact of TeamSTEPPS + tailored implementation strategies on inter-professional collaboration, teamwork, and student outcomes. In the future, we hope to explore whether TeamSTEPPS may be flexibly used to improve current school mental health services as a pre-implementation strategy to improve a system’s readiness to adopt a youth mental health evidence-based program and/or as an adjunct intervention during evidence-based program implementation to increase the probability of adoption.

### Conclusion

TeamSTEPPS is a promising strategy to improve team work-related processes in school settings as part of an effort to improve the quality of student mental health. Working alongside diverse school partners to adapt the intervention and develop a tailored implementation plan is in line with best practices for adapting interventions to new settings. Future studies will report on primary outcomes of this multi-year implementation effort.

## Supplementary Information

Below is the link to the electronic supplementary material.

Supplementary file1 (DOCX 457 kb)

## References

[CR1] Aarons, G. A., Ehrhart, M. G., Moullin, J. C., Torres, E. M., & Green, A. E. (2017). Testing the leadership and organizational change for implementation (LOCI) intervention in substance abuse treatment: A cluster randomized trial study protocol. *Implementation Science,**12*(1), 29. 10.1186/s13012-017-0562-3. 28253900 10.1186/s13012-017-0562-3 PMC5335741

[CR2] Agency for Healthcare Research and Quality. (n.d.) *TeamSTEPPS*^*®*^* team strategies & tools to**enhance performance & patient safety*. Retrieved July 21, 2023, from https://www.ahrq.gov/teamstepps-program/index.html. 21249942

[CR3] Bates, S. M., Mellin, E., Paluta, L. M., Anderson-Butcher, D., Vogeler, M., & Sterling, K. (2019). Examining the influence of interprofessional team collaboration on student-level outcomes through school-community partnerships. *Children & Schools,**41*(2), 111–122. 10.1093/cs/cdz001.

[CR7] Bohnenkamp, J. H., Patel, C., Connors, E., Orenstein, S., Ereshefsky, S., Lever, N., & Hoover, S. (2023). Evaluating strategies to promote effective, multidisciplinary team collaboration in school mental health. *Journal of Applied School Psychology,**39*(2), 130–150. 10.1080/15377903.2022.2077875. 37207133 10.1080/15377903.2022.2077875 PMC10195065

[CR8] Briesch, A. M., Cintron, D. W., Dineen, J. N., Chafouleas, S. M., McCoach, D. B., & Auerbach, E. (2020). Comparing stakeholders’ knowledge and beliefs about supporting students’ social, emotional, and behavioral health in schools. *School Mental Health,**12*(2), 222238. 10.1007/s12310-019-09355-9.

[CR9] Brookman-Frazee, L., Chlebowski, C., Suhrheinrich, J., Finn, N., Dickson, K. S., Aarons, G. A., & Stahmer, A. (2020). Characterizing shared and unique implementation influences in two community services systems for autism: Applying the EPIS framework to two large scale autism intervention community effectiveness trials. *Administration and Policy in Mental Health and Mental Health Services Research,**47*(2), 176–187. 10.1007/s10488-019-00931-4. 30905009 10.1007/s10488-019-00931-4 PMC6756990

[CR10] Chen, A. S., Yau, B., Revere, L., & Swails, J. (2019). Implementation, evaluation, and outcome of TeamSTEPPS in interprofessional education: A scoping review. *Journal of Interprofessional Care,**33*(6), 795–804. 10.1080/13561820.2019.1594729. 31009273 10.1080/13561820.2019.1594729

[CR11] Connors, E. H., Smith-Millman, M., Bohnenkamp, J. H., Carter, T., Lever, N., & Hoover, S. A. (2020). Can we move the needle on school mental health quality through systematic quality improvement collaboratives? *School Mental Health,**12*(3), 478–492. 10.1007/s12310-020-09374-x. 34322180 10.1007/s12310-020-09374-x PMC8315192

[CR12] Cook, C. R., Lyon, A. R., Locke, J., Waltz, T., & Powell, B. J. (2019). Adapting a compilation of implementation strategies to advance school-based implementation research and practice. *Prevention Science,**20*(6), 914–935. 10.1007/s11121-019-01017-1. 31152328 10.1007/s11121-019-01017-1 PMC8943907

[CR13] Costello, E. J., He, J., Sampson, N. A., Kessler, R. C., & Merikangas, K. R. (2014). Services for adolescent psychiatric disorders: 12-month data from the National Comorbidity Survey-Adolescent. *Psychiatric Services (washington, d.c.),**65*(3), 359–366. 10.1176/appi.ps.201100518. 24233052 10.1176/appi.ps.201100518 PMC4123755

[CR14] Damschroder, L. J., Aron, D. C., Keith, R. E., Kirsh, S. R., Alexander, J. A., & Lowery, J. C. (2009). Fostering implementation of health services research findings into practice: A consolidated framework for advancing implementation science. *Implementation Science,**4*(1), 50. 10.1186/1748-5908-4-50. 19664226 10.1186/1748-5908-4-50 PMC2736161

[CR15] Dimitropoulos, G., Cullen, E., Cullen, O., Pawluk, C., McLuckie, A., Patten, S., Bulloch, A., Wilcox, G., & Arnold, P. D. (2022). “Teachers often see the red flags first”: Perceptions of school staff regarding their roles in supporting students with mental health concerns. *School Mental Health,**14*(2), 402–415. 10.1007/s12310-021-09475-1.

[CR16] Duong, M. T., Bruns, E. J., Lee, K., Cox, S., Coifman, J., Mayworm, A., & Lyon, A. R. (2021). Rates of mental health service utilization by children and adolescents in schools and other common service settings: A systematic review and meta-analysis. *Administration and Policy in Mental Health and Mental Health Services Research,**48*(3), 420–439. 10.1007/s10488-020-01080-9. 32940884 10.1007/s10488-020-01080-9

[CR17] Fabiano, G. A., & Evans, S. W. (2019). Introduction to the special issue of School Mental Health on best practices in effective multi-tiered intervention frameworks. *School Mental Health: A Multidisciplinary Research and Practice Journal,**11*(1), 1–3. 10.1007/s12310-018-9283-2.

[CR18] Farmer, E. M. Z., Burns, B. J., Phillips, S. D., Angold, A., & Costello, E. J. (2003). Pathways into and through mental health services for children and adolescents. *Psychiatric Services (washington, d.c.),**54*(1), 60–66. 10.1176/appi.ps.54.1.60. 12509668 10.1176/appi.ps.54.1.60

[CR19] Frank, H. E., Saldana, L., Kendall, P. C., Schaper, H. A., & Norris, L. A. (2022). Bringing evidence-based interventions into the schools: An examination of organizational factors and implementation outcomes. *Child & Youth Services,**43*(1), 28–52. 10.1080/0145935x.2021.1894920. 35814495 10.1080/0145935x.2021.1894920 PMC9268029

[CR20] Heatly, M. C., Nichols-Hadeed, C., Stiles, A. A., & Alpert-Gillis, L. (2023). Implementation of a school mental health learning collaborative model to support cross-sector collaboration. *School Mental Health,**15*(2), 384–401. 10.1007/s12310023-09578-x. 10.1007/s12310-023-09578-x PMC10102686 37359161

[CR21] Hjörne, E., & Säljö, R. (2014). Analysing and preventing school failure: Exploring the role of multi-professionality in pupil health team meetings. *International Journal of Educational Research,**63*, 5–14. 10.1016/j.ijer.2012.09.005.

[CR22] Holmesland, A.-L., Seikkula, J., Nilsen, Ø., Hopfenbeck, M., & Arnkil, T. E. (2010). Open dialogues in social networks: Professional identity and transdisciplinary collaboration. *International Journal of Integrated Care*. 10.5334/ijic.564. 20922064 10.5334/ijic.564 PMC2948679

[CR23] Hoover, S. A. (2018). When we know better, we don’t always do better: Facilitating the research to practice and policy gap in school mental health. *School Mental Health,**10*(2), 190–198. 10.1007/s12310-018-9271-6.

[CR24] Kamali, M., Edwards, J., Anderson, L. N., Duku, E., & Georgiades, K. (2022). Social disparities in mental health service use among children and youth in Ontario: evidence from a general, population-based survey. *Canadian Journal of Psychiatry*. 10.1177/07067437221144630. 36503305 10.1177/07067437221144630 PMC10411367

[CR25] Kilbourne, A. M., Neumann, M. S., Pincus, H. A., Bauer, M. S., & Stall, R. (2007). Implementing evidence-based interventions in health care: Application of the replicating effective programs framework. *Implementation Science,**2*(1), 42. 10.1186/1748-5908-2-42. 18067681 10.1186/1748-5908-2-42 PMC2248206

[CR26] Kilbourne, A. M., Smith, S. N., Choi, S. Y., Koschmann, E., Liebrecht, C., Rusch, A., Abelson, J. L., Eisenberg, D., Himle, J. A., Fitzgerald, K., & Almirall, D. (2018). Adaptive school based implementation of CBT (ASIC): Clustered-SMART for building an optimized adaptive implementation intervention to improve uptake of mental health interventions in schools. *Implementation Science,**13*(1), 119. 10.1186/s13012-018-0808-8. 30185192 10.1186/s13012-018-0808-8 PMC6126013

[CR27] King, H. B., Battles, J., Baker, D. P., Alonso, A., Salas, E., Webster, J., Toomey, L., & Salisbury, M. (2008). TeamSTEPPSTM: Team strategies and tools to enhance performance and patient safety. In K. Henriksen, J. B. Battles, M. A. Keyes, & M. L. Grady (Eds.), *Advances in patient safety: New directions and alternative approaches. * (Vol. 3: Performance and Tools). Agency for Healthcare Research and Quality (US). 21249942

[CR28] Kirk, M. A., Kelley, C., Yankey, N., Birken, S. A., Abadie, B., & Damschroder, L. (2016). A systematic review of the use of the consolidated framework for implementation research. *Implementation Science,**11*(1), 72. 10.1186/s13012-016-0437-z. 27189233 10.1186/s13012-016-0437-z PMC4869309

[CR6] Kuriyan, A., Kinkler, G., Cidav, Z., Kang-Yi, C., Eiraldi, R., Salas, E., & Wolk, C.B. (2021). Team strategies and tools to enhance performance and patient safety (TeamSTEPPS) to improve collaboration in school mental health: Protocol for a mixed methods hybrid effectiveness-implementation study. *JMIR Research Protocols,**10*(2), e26567. 10.2196/26567. 33555258 10.2196/26567 PMC7899798

[CR29] Locke, J., Kang-Yi, C., Frederick, L., & Mandell, D. S. (2020). Individual and organizational characteristics predicting intervention use for children with autism in schools. *Autism,**24*(5), 1152–1163. 10.1177/1362361319895923. 31867987 10.1177/1362361319895923 PMC7308214

[CR30] Lyon, A. R., & Bruns, E. J. (2019). From evidence to impact: joining our best school mental health practices with our best implementation strategies. *School Mental Health,**11*(1), 106–114. 10.1007/s12310-018-09306-w. 31709018 10.1007/s12310-018-09306-w PMC6839825

[CR31] Lyon, A. R., Liu, F. F., Connors, E. H., King, K. M., Coifman, J. I., Cook, H., McRee, E., Ludwig, K., Law, A., Dorsey, S., & McCauley, E. (2022). How low can you go? Examining the effects of brief online training and post-training consultation dose on implementation mechanisms and outcomes for measurement-based care. *Implementation Science Communications,**3*(1), 79. 10.1186/s43058-022-00325-y. 35869500 10.1186/s43058-022-00325-y PMC9306246

[CR32] Mahoney, J. S., Ellis, T. E., Garland, G., Palyo, N., & Greene, P. K. (2012). Supporting a psychiatric hospital culture of safety. *Journal of the American Psychiatric Nurses Association,**18*(5), 299–306. 10.1177/1078390312460577. 22967939 10.1177/1078390312460577

[CR33] Mayer, C. M., Cluff, L., Lin, W.-T., Willis, T. S., Stafford, R. E., Williams, C., Saunders, R., Short, K. A., Lenfestey, N., Kane, H. L., & Amoozegar, J. B. (2011). Evaluating efforts to optimize TeamSTEPPS implementation in surgical and pediatric intensive care units. *The Joint Commission Journal on Quality and Patient Safety,**37*(8), 365-AP3. 10.1016/S1553-7250(11)37047-X. 21874972 10.1016/s1553-7250(11)37047-x

[CR34] McGuier, E. A., Feldman, J., Bay, M., Ascione, S., Tatum, M., Salas, E., & Kolko, D. J. (2023). Improving teamwork in multidisciplinary cross-sector teams: Adaption and pilot testing of a team training for Child Advocacy Center teams. *Children and Youth Services Review,**153*, 107096. 10.1016/j.childyouth.2023.107096. 37601235 10.1016/j.childyouth.2023.107096 PMC10437145

[CR35] McQuillin, S. D., Lyons, M. D., Becker, K. D., Hart, M. J., & Cohen, K. (2019). Strengthening and expanding child services in low resource communities: The role of task-shifting and just-in-time training. *American Journal of Community Psychology,**63*(3–4), 355365. 10.1002/ajcp.12314. 10.1002/ajcp.12314 30834554

[CR36] Mendenhall, A., Iachini, A., & Anderson-Butcher, D. (2013). Exploring stakeholder perceptions of facilitators and barriers to implementation of an expanded school improvement model. *Children & Schools,**35*, 225–234. 10.1093/cs/cdt011.

[CR37] Moore, G., Campbell, M., Copeland, L., Craig, P., Movsisyan, A., Hoddinott, P., Littlecott, H., O’Cathain, A., Pfadenhauer, L., Rehfuess, E., Segrott, J., Hawe, P., Kee, F., Couturiaux, D., Hallingberg, B., & Evans, R. (2021). Adapting interventions to new contexts—The ADAPT guidance. *BMJ,**374*, n1679. 10.1136/bmj.n1679. 34344699 10.1136/bmj.n1679 PMC8329746

[CR38] New Jersey Department of Education. (n.d.a). *2021–2022 certificated staff.* Retrieved April 26, 2023, from https://www.nj.gov/education/doedata/cs/.

[CR39] New Jersey Department of Education. (n.d.b). *2021–2022 enrollment district reported data.* Retrieved April 26, 2023, from https://www.nj.gov/education/doedata/enr/.

[CR40] Ødegård, A. (2005). Perceptions of interprofessional collaboration in relation to children with mental health problems. A pilot study. *Journal of Interprofessional Care,**19*(4), 347–357. 10.1080/13561820500148437. 16076596 10.1080/13561820500148437

[CR41] Olson, J. R., Lucy, M., Kellogg, M. A., Schmitz, K., Berntson, T., Stuber, J., & Bruns, E. J. (2021). What happens when training goes virtual? Adapting training and technical assistance for the school mental health workforce in response to COVID-19. *School Mental Health: A Multidisciplinary Research and Practice Journal,**13*(1), 160–173. 10.1007/s12310-020-09401-x. 10.1007/s12310-020-09401-x PMC7781169 33425042

[CR42] Pennsylvania Department of Education. *2021–22 professional staff summary*. (n.d.a). Retrieved April 26, 2023, from https://www.education.pa.gov/DataAndReporting/ProfSupPers/Pages/ProfStaffSummary.aspx.

[CR43] Pennsylvania Department of Education. *2021–2022 PA lunch report* (n.d.b). Retrieved April 26, 2023, from https://www.education.pa.gov/Teachers%20-%20Administrators/Food-Nutrition/reports/Pages/National-School-Lunch-Program-Reports.aspx.

[CR44] Pennsylvania Department of Education. *Public school enrollment 2021–2022*. (n.d.c). Retrieved April 26, 2023, from https://www.education.pa.gov:443/DataAndReporting/Enrollment/Pages/PublicSchEnrReports.aspx.

[CR45] Reaves, S., Bohnenkamp, J., Mayworm, A., Sullivan, M., Connors, E., Lever, N., Kelly, M. S., Bruns, E. J., & Hoover, S. (2022). Associations between school mental health team membership and impact on service provision. *School Mental Health,**14*(3), 672–684. 10.1007/s12310-021-09493-z. 35003376 10.1007/s12310-021-09493-z PMC8729097

[CR46] Reuterswärd, M., & Hylander, I. (2017). Shared responsibility: School nurses’ experience of collaborating in school-based interprofessional teams. *Scandinavian Journal of Caring Sciences,**31*(2), 253–262. 10.1111/scs.12337. 27440472 10.1111/scs.12337

[CR47] Richter, A., Sjunnestrand, M., Romare Strandh, M., & Hasson, H. (2022). Implementing school-based mental health services: A scoping review of the literature summarizing the factors that affect implementation. *International Journal of Environmental Research and Public Health,**19*(6), 3489. 10.3390/ijerph19063489. 35329175 10.3390/ijerph19063489 PMC8948726

[CR48] Sheppard, F., Williams, M., & Klein, V. R. (2013). TeamSTEPPS and patient safety in healthcare. *Journal of Healthcare Risk Management,**32*(3), 5–10. 10.1002/jhrm.21099. 23335296 10.1002/jhrm.21099

[CR49] Smith, S. N., Almirall, D., Choi, S. Y., Koschmann, E., Rusch, A., Bilek, E., Lane, A., Abelson, J. L., Eisenberg, D., Himle, J. A., Fitzgerald, K. D., Liebrecht, C., & Kilbourne, A. M. (2022). Primary aim results of a clustered SMART for developing a school-level, adaptive implementation strategy to support CBT delivery at high schools in Michigan. *Implementation Science,**17*(1), 42. 10.1186/s13012-022-01211-w. 35804370 10.1186/s13012-022-01211-w PMC9264291

[CR50] Southam-Gerow, M. A., Hourigan, S. E., & Allin, R. B. (2009). Adapting evidence-based mental health treatments in community settings: Preliminary results from a partnership approach. *Behavior Modification,**33*(1), 82–103. 10.1177/0145445508322624. 18697917 10.1177/0145445508322624 PMC2674398

[CR51] Splett, J. W., Perales, K., Halliday-Boykins, C. A., Gilchrest, C. E., Gibson, N., & Weist, M. D. (2017). Best practices for teaming and collaboration in the interconnected systems framework. *Journal of Applied School Psychology,**33*(4), 347–368. 10.1080/15377903.2017.1328625.

[CR52] Stead, K., Kumar, S., Schultz, T. J., Tiver, S., Pirone, C. J., Adams, R. J., & Wareham, C. A. (2009). Teams communicating through STEPPS. *The Medical Journal of Australia,**190*(S11), S128–S132. 10.5694/j.1326-5377.2009.tb02619.x. 19485861 10.5694/j.1326-5377.2009.tb02619.x

[CR59] Stirman, S.W., Baumann, A. A., & Miller, C. J. (2019). The FRAME: An expanded framework for reporting adaptations and modifications to evidence-based interventions. *Implementation Science*, *14*(1), 58. 10.1186/s13012-019-0898-y 31171014 10.1186/s13012-019-0898-y PMC6554895

[CR53] Taylor, B., Henshall, C., Kenyon, S., Litchfield, I., & Greenfield, S. (2018). Can rapid approaches to qualitative analysis deliver timely, valid findings to clinical leaders? A mixed methods study comparing rapid and thematic analysis. *British Medical Journal Open,**8*(10), e019993. 10.1136/bmjopen-2017-019993. 10.1136/bmjopen-2017-019993 PMC6194404 30297341

[CR54] Wang, K., Chen, Y., Zhang, J., & Oudekerk, B. (2020). *Indicators of school crime and safety: 2019*. National Center for Education Statistics & Bureau of Justice Statistics. https://nces.ed.gov/pubs2020/2020063.pdf.

[CR55] Weist, M. D., Mellin, E. A., Chambers, K. L., Lever, N. A., Haber, D., & Blaber, C. (2012). Challenges to collaboration in school mental health and strategies for overcoming them. *The Journal of School Health,**82*(2), 97–105. 10.1111/j.1746-1561.2011.00672.x. 22239135 10.1111/j.1746-1561.2011.00672.x

[CR56] Whitaker, K., Fortier, A., Bruns, E. J., Nicodimos, S., Ludwig, K., Lyon, A. R., Pullmann, M. D., Short, K., & McCauley, E. (2018). How do school mental health services vary across contexts? Lessons learned from two efforts to implement a research-based strategy. *School Mental Health: A Multidisciplinary Research and Practice Journal,**10*(2), 134–146. 10.1007/s12310-017-9243-2.

[CR57] Williams, C. K., Strickland, A. L., Riggs, N. R., Dyett, A. R., Gibson, Z. R., & Pulskamp, A. D. (2018). Colorado healthy schools smart source: Testing the association between collaboration with community mental health centers and tier 2 implementation. *School Mental Health,**10*(2), 163–172. 10.1007/s12310-018-9247-6.

[CR4] Wolk, C. B., Stewart, R. E., Cronholm, P., Eiraldi, R., Salas, E., & Mandell, D.S. (2019a). Adapting TeamSTEPPS for school mental health teams: A pilot study. *Pilot and Feasibility Studies,**5*(1), 148. 10.1186/s40814-019-0529z. 31890260 10.1186/s40814-019-0529-z PMC6918659

[CR5] Wolk, C. B., Stewart, R. E., Eiraldi, R., Cronholm, P., Salas, E., & Mandell, D. S. (2019b). The implementation of a team training intervention for school mental health: Lessons learned. *Psychotherapy,**56*(1), 83–90. 10.1037/pst0000179. 30489095 10.1037/pst0000179 PMC6395502

[CR58] Zabek, F., Lyons, M. D., Alwani, N., Taylor, J. V., Brown-Meredith, E., Cruz, M. A., & Southall, V. H. (2023). Roles and functions of school mental health professionals within comprehensive school mental health systems. *School Mental Health,**15*(1), 1–18. 10.1007/s12310-022-09535-0. 35911088 10.1007/s12310-022-09535-0 PMC9321305

