# Supplementary material for: Partnering with Schools to Adapt a Team Science Intervention: Processes and Challenges
Source: School Ment Health. 2024 May 2;16(3):695–709. doi: 10.1007/s12310-024-09665-7 (PMC11452467; doi:10.1007/s12310-024-09665-7)

**Supplemental Material 1.**

TeamSTEPPS Advisory Board Agenda (Abbreviated)

| **Day 1 Agenda** | |
| --- | --- |
| **Topic** | **Activity** |
| Welcome, overview of Day 1, establish norms and expectations for meetings | Introductions |
| Overview of TeamSTEPPS model | Didactic |
| Initial reactions to TeamSTEPPS model | Discussion |
| TeamSTEPPS Domain: Team structure | Create organizational chart of your team |
| TeamSTEPPS Domain: Leadership | Discuss current leadership plan, challenges, and potential solutions |
| TeamSTEPPS Domain: Mutual support | Discuss current mutual support plan, challenges, and potential solutions |
| TeamSTEPPS Domain: Communication | Discuss current communication plan, challenges, and potential solutions |
| TeamSTEPPS Domain: Situation Monitoring | Discuss current situation monitoring plan, challenges, and potential solutions |
| Summary, Preview of Day 2 | Q&A |

**Key questions to answer on Day 1:**

- Whose perspectives are missing, and how can their input be obtained?
- What TeamSTEPPS strategies are already being used by the team?
- How can team members see these strategies getting used on their team, if at all?
- What situations tend to create potential problems or disagreements?
- What are some potential solutions to common problems faced by the team, and how can TeamSTEPPS strategies be used in these situations?
- Who is on your team? Is there any overlap in roles? How do team members know what tasks they are responsible for? Do any of your current procedures outline who is responsible for what?
- What do team meetings currently look like? Are there processes in place for problem-solving in the moment, debriefing after meetings or events, etc.?
- How and when does your team currently communicate? When does communication tend to break down?

**Supplemental Material 1 continued.**

| **Day 2 Agenda** | |
| --- | --- |
| **Topic** | **Activity** |
| Welcome, recap of Day 1, overview of Day 2 | Didactic |
| Assessing potential barriers to implementation of TeamSTEPPS | 1. Identify barriers to implementing TeamSTEPPS* 2. Sort barriers by four quadrants (likelihood of event occurring, how devastating it would be)*   Once complete, identify top 3 most significant challenges |
| Reviewing potential solutions to implementation of TeamSTEPPS | Small groups to brainstorm solutions to top three barriers identified from previous activity, then discuss as larger group |
| Summary, expectations for next steps | Q & A |

*Pictured below. We utilized Google Jamboard for this activity.

**Key questions to answer on Day 2:**

- Which specific team members will be responsible for implementing TeamSTEPPS?
- What will implementation and sustainment look like beyond initial training sessions?
- How will TeamSTEPPS be integrated into onboarding process for new hires (e.g., web-based training)?
- How will TeamSTEPPS become part of the school culture?
- How will the school school identify and support a TeamSTEPPS champion?
- What outcome variables would measure whether TeamSTEPPS is successful? How will this data be collected over time?

**Supplemental Material 2.**

Example Advisory Board Meeting Products


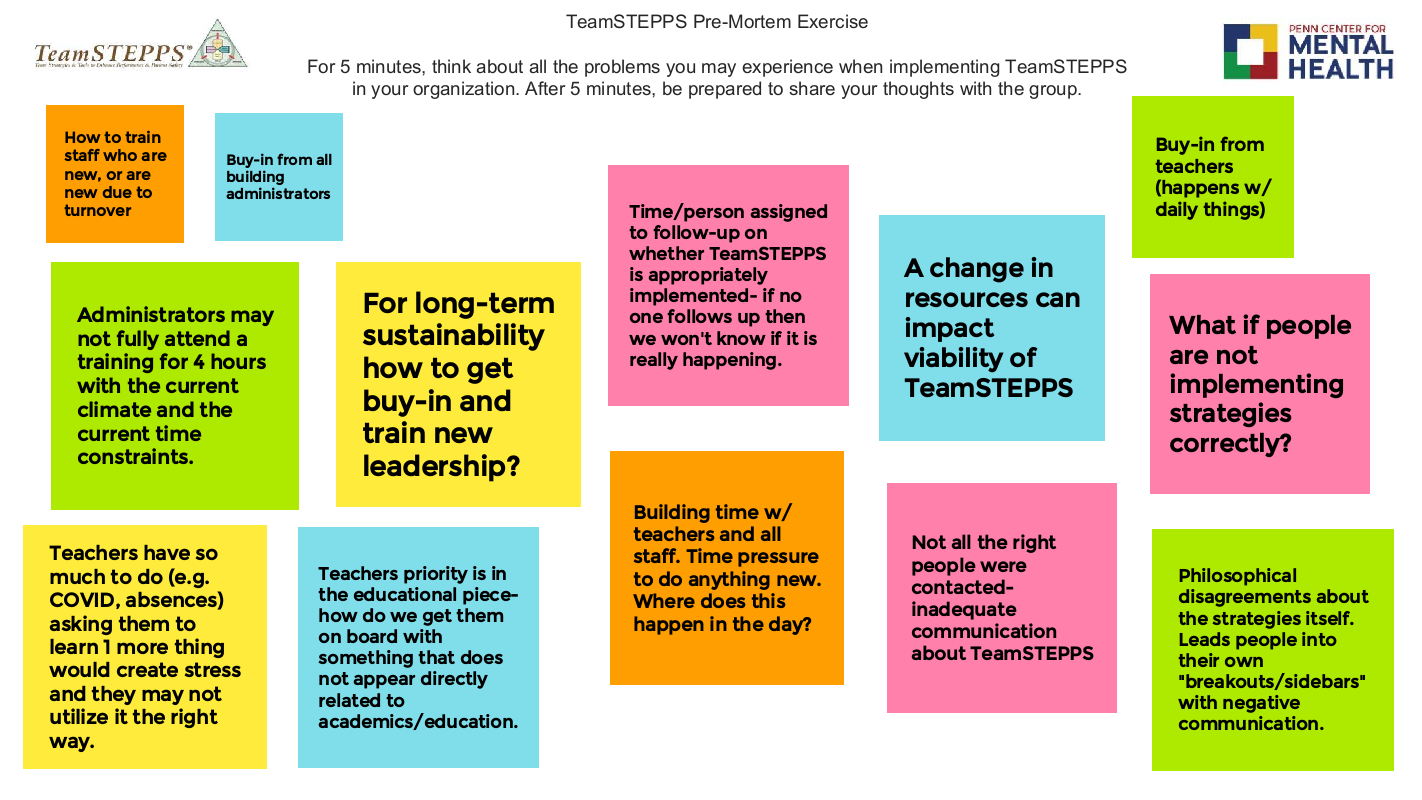


**Supplemental Material 2 continued.**


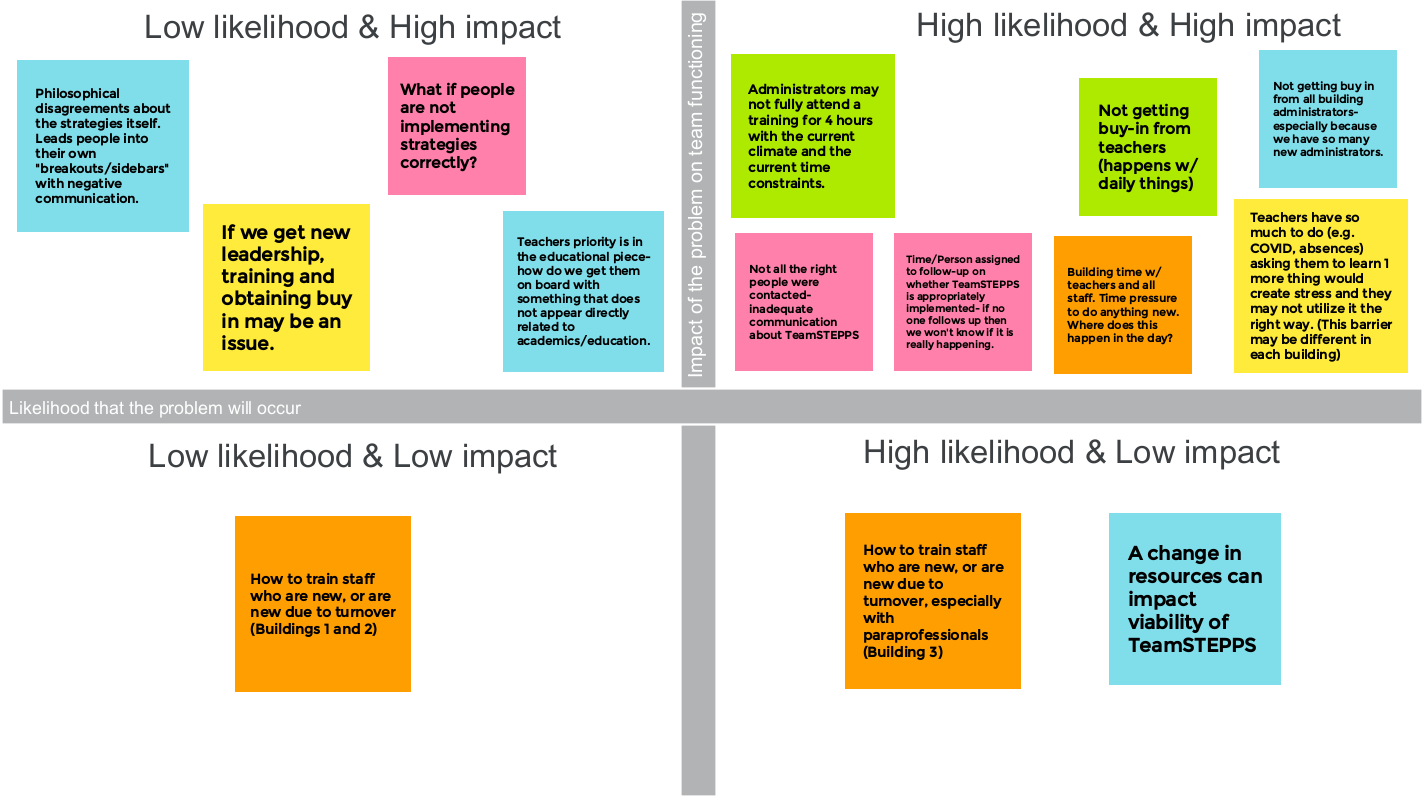

Supplement: Supplementary file 1 — Supplementary file1 (DOCX 457 kb) [file 12310_2024_9665_MOESM1_ESM.docx]
